# Instantaneous Cure of Whooping Cough

**Published:** 1889-11

**Authors:** M. Hartwig

**Affiliations:** 38 East Huron street


*Editors Buffalo Medical and Surgical Journal*:

Some statements reappear yearly like the sea-serpent, for instance the instantaneous cure of whooping-cough by Mohn's proceeding with sulphurous acid fumigations. Your esteemed JOURNAL, page 173, of October, 1889, repeats once more Mohn's statement. May be there is truth at the bottom of it, but that this proceeding is *always* successful is far from true. Young practitioners would be misled, hence my refutation based on actual careful trial. If others, Schoenberg, in Christiania, excepted, would have had results like Mohn, this would be now the only method of treating whooping cough, but it is not.

Very respectfully, M. HARTWIG.

38 EAST HURON STREET, October 1, 1889.

---

### HUMOR.

Old Mrs. Smiley : "Next time I get took down sick, my dear, I wish ye wouldn't have that there young sprig of a doctor come to attend me. I don't go much on young doctors, no how." Mr. Smiley : "Well, Maria, who would you like to have me call ?" Mrs. S. : "I've kinder took a notion to the doctor around the corner. I dun no much about him ; but I see he's got a sign out 'Veterinary Surgeon,' and I think he must be a man of experience."—*Cincinnati Lancet-Clinic.*

